# Response to: The Dark Side of 3D Simulation in Breast Augmentation: How to Use Its Advantages and Avoid Its Drawbacks

**DOI:** 10.1093/asj/sjaf016

**Published:** 2025-01-30

**Authors:** Isil Akgun Demir

I appreciate Montemurro et al's insights into 3-dimensional (3D) breast simulation, as they significantly contribute to our understanding of the negative aspects and major drawbacks of this tool.^1^ I would like to express my support for their perspective and offer some additional comments and considerations regarding the points raised.

As clearly described by the authors, 3D simulation tools should be avoided in patients with “out-of-the-box” anatomies, including asymmetries of both breasts and thorax. Utilizing these tools in such cases often results in misleading and unsatisfactory images that may demotivate patients. Instead, this method should be used for patient education, illustrating existing asymmetries and projection differences, while explaining the need for different implant sizes and the potential for achieving greater symmetry through asymmetric implant placement.

Another significant disadvantage is the failure to demonstrate how fat grafting can help address existing asymmetries, which cannot be adequately managed by simply selecting different implants. Additionally, the limitations of the simulation in showing improvements in the upper pole transition are notable.

The phrase “once they see it, they cannot unsee it” aptly describes the experience of patients using 3D simulation who possess challenging anatomical features, such as wide intermammary distance, tuberous breasts, ptosis, thorax abnormalities, and unusual nipple–areola complex (NAC) structures. As the software adjusts images primarily through volume changes, it does not accurately reflect the stretching effect of the implant on the areola, nor does it illustrate the changes in NAC position and other surgical maneuvers that would be employed to optimize outcomes. For instance, the use of anatomical implants to elevate the NAC cannot be effectively demonstrated; 3D simulations that depict NACs as positioned inferiorly can leave patients with a negative impression of potential surgical results. In terms of fostering a healthy patient–surgeon relationship, it may be beneficial to intentionally skip this aspect of the consultation in certain cases.

Furthermore, because 3D simulation programs allow surgeons to place larger implant sizes while still producing reasonably appealing results—similar to external sizers—patients may be left with the impression that they can request implants as large as they desire. Therefore, surgeons must caution patients about physical limitations and refrain from permitting excessive requests during try-ons. Even for the most suitable candidates, it is essential to continuously remind patients that simulations are not 100% accurate.

3D simulation becomes a valuable tool when used to support clinical assessment. Physical measurements, including breast width and tissue thickness, remain the mainstay for implant selection. In this context, 3D simulation and external sizers serve as supplementary measures to facilitate patients’ understanding of surgical outcomes.

Once again, I would like to congratulate the authors for their thoughtful work on “The Dark Side of 3D Simulation in Breast Augmentation: How to Use Its Advantages and Avoid Its Drawbacks,” and I express my gratitude to the editor for the opportunity to share my insights.
